# Ion-induced surface reactions and deposition from Pt(CO)_2_Cl_2_ and Pt(CO)_2_Br_2_

**DOI:** 10.3762/bjnano.15.115

**Published:** 2024-11-19

**Authors:** Mohammed K Abdel-Rahman, Patrick M Eckhert, Atul Chaudhary, Johnathon M Johnson, Jo-Chi Yu, Lisa McElwee-White, D Howard Fairbrother

**Affiliations:** 1 Department of Chemistry, Johns Hopkins University, Baltimore, MD, 21218, USA; 2 Department of Chemistry, University of Florida, Gainesville, Florida, 32611-7200, USA

**Keywords:** deposition, ion beam, nanostructure, organometallic, precursor

## Abstract

Ion beam-induced deposition (IBID) using Pt(CO)_2_Cl_2_ and Pt(CO)_2_Br_2_ as precursors has been studied with ultrahigh-vacuum (UHV) surface science techniques to provide insights into the elementary reaction steps involved in deposition, complemented by analysis of deposits formed under steady-state conditions. X-ray photoelectron spectroscopy (XPS) and mass spectrometry data from monolayer thick films of Pt(CO)_2_Cl_2_ and Pt(CO)_2_Br_2_ exposed to 3 keV Ar^+^, He^+^, and H_2_^+^ ions indicate that deposition is initiated by the desorption of both CO ligands, a process ascribed to momentum transfer from the incident ion to adsorbed precursor molecules. This precursor decomposition step is accompanied by a decrease in the oxidation state of the Pt(II) atoms and, in IBID, represents the elementary reaction step that converts the molecular precursor into an involatile PtX_2_ species. Upon further ion irradiation these PtCl_2_ or PtBr_2_ species experience ion-induced sputtering. The difference between halogen and Pt sputter rates leads to a critical ion dose at which only Pt remains in the film. A comparison of the different ion/precursor combinations studied revealed that this sequence of elementary reaction steps is invariant, although the rates of CO desorption and subsequent physical sputtering were greatest for the heaviest (Ar^+^) ions. The ability of IBID to produce pure Pt films was confirmed by AES and XPS analysis of thin film deposits created by Ar^+^/Pt(CO)_2_Cl_2_, demonstrating the ability of data acquired from fundamental UHV surface science studies to provide insights that can be used to better understand the interactions between ions and precursors during IBID from inorganic precursors.

## Introduction

Focused ion beam-induced deposition (FIBID) and focused electron beam-induced deposition (FEBID) are vacuum-based, charged-particle bottom-up nanofabrication techniques that directly fabricate metal containing nanostructures as a consequence of the reactions between ions or electrons and organometallic precursors that are transiently adsorbed on a substrate surface [1–6]. Charged-particle-induced deposition techniques offer control over process parameters such as particle position, energy, beam current, and flux, allowing for the formation of nanoscale patterns. Since they are direct-write techniques, they do not require the use of organic solvents present in traditional lithography. Indeed, FEBID/FIBID can be considered as alternatives to commonly used methods such as chemical vapor deposition (CVD) and atomic layer deposition (ALD), particularly for area-selective, as opposed to conformal, deposition strategies in various applications, such as circuit editing and lithographic mask repair in the semiconductor industry [7–12] as well as the growth of functional materials for magnetism [13–16], superconductivity [17], and sensing [18–19]. Compared to FEBID, FIBID operates with higher current densities leading to more rapid deposition rates [20], creates deposits with metal contents that are typically higher than those observed in FEBID and has a wider choice of charged-particle sources [21–25].

One of the major disadvantages of charged-particle deposition techniques is that they produce deposits with relatively low metal contents as compared to CVD and ALD. Indeed, creating deposits with high metal contents is one of the biggest challenges for FIBID and FEBID, and recent advances in precursor design and fine-tuning of deposition parameters led to deposits of greater purity such as the FEBID fabrication of Co_3_Fe nanowires [15,26–27]. Similarly, the frequently studied Pt precursor MeCpPtMe_3_ yields a deposit with nearly 100% Pt content from CVD [28–29]. This purity is achieved by utilizing reactive carrier gases, such as H_2_ and O_2_, as co-reactants during depositions at elevated temperatures to induce Pt deposition and to remove carbon by hydrogenation to volatile hydrocarbons or oxidation to form H_2_O, CO, and CO_2_ as volatile products. In contrast, this precursor results in Pt purities of less than 20% in FEBID [30–31], while FIBID has been shown to provide deposits with greater Pt content than FEBID expressed by C/Pt ratios of 4:1 or lower [21,32–33]. FIBID is commonly conducted using Ga^+^ ion sources; however, ion implantation is observed with gallium contamination reported between 5% [34] and 30% [33] in the resulting deposits. Ion implantation can be largely avoided by using noble gas ions like He^+^ [32,35] or Ar^+^ [21], which are commonly used in helium ion microscopes and focused ion beam milling instruments.

In FIBID, ion-induced interactions can initiate a complex mixture of different processes including ion-induced deposition, secondary electron emission, and physical sputtering of adsorbed or substrate atoms [21–22,25,31,36–40]. Ion-induced deposition can occur via a momentum/energy transfer process [21,25,41–42] that results in the decomposition of the precursor to form volatile species and an involatile deposit containing the metal of interest. Furthermore, as the volatile species escape the system, they can collide with adsorbed material leading to a cascade of momentum transfer events [43]. In contrast to FIBID, FEBID occurs via different electron stimulated mechanisms, namely, dissociative electron attachment (DEA), dissociative ionization (DI), neutral dissociation (ND), and dipolar dissociation (DD) [44–52].

One of the most important factors that govern deposit purity is the identity of ligands present in the precursor. The coordination sphere of ligands is addressed by precursor design [5,53–54]. An ideal precursor candidate would have sufficient volatility and stability for the transport of intact gas phase precursor molecules during the process. The ligands should readily and cleanly be liberated from the precursor upon irradiation to provide a metallic deposit in the path of the ion beam. For Pt deposits, although MeCpPtMe_3_ meets the volatility and stability requirements, the inability to directly desorb the cyclopentadienyl ligands results in significant carbon contamination upon ion irradiation [21]. Alternatives to the carbon-rich MeCpPtMe_3_ complex are the four coordinate Pt(CO)_2_X_2_ complexes (X = Cl, Br). These precursors are attractive because they contain a larger Pt content (14 atom %) compared to MeCpPtMe_3_ (C_9_H_16_Pt, 4 atom %). Furthermore, CO is known to readily desorb from metal carbonyls upon electron- or ion-beam irradiation [22–25,45,55–56]. Indeed, Pt(CO)_2_Br_2_ [55] and Pt(CO)_2_Cl_2_ [55,57] have been explored as FEBID precursors. The compositions of deposits from these precursors varied with the deposition conditions. Under UHV conditions, the deposits were carbon-free but contained significant halogen contamination, which could be removed only after prolonged electron irradiation by means of electron-stimulated desorption and only if the films were sufficiently (nanometer scale) thin. In contrast, under high-vacuum conditions found in scanning electron microscopes, the deposits were halogen-free but contained carbon contamination [57]. These deposits from Pt(CO)_2_Cl_2_ and Pt(CO)_2_Br_2_ resulted in deposits with Pt purities comparable to deposits made from MeCpPtMe_3_ [55,58]. Although the Pt content was similar, the deposits made from Pt(CO)_2_X_2_ suffered from a slower growth rate than those from MeCpPtMe_3_; this difference is attributed to the lower precursor partial pressures of Pt(CO)_2_Cl_2_ and Pt(CO)_2_Br_2_ [55]. A related precursor for Ru deposition, Ru(CO)_4_I_2_, was the subject of a UHV/FIBID study where deposition occurred via Ar^+^-induced removal of the CO ligands of the precursor, which was followed by physical sputtering of I from residual RuI_2_ and slower sputtering of Ru [23].

Another important factor unique to FIBID that governs deposit purity can be the chemical identity of the ion [33,59–64]. Interactions between ions and adsorbed precursors are momentum-driven, which results in the formation of nonvolatile deposits as well as sputtering of deposited material [21–22,25]. Recently, it was reported that the ion identity plays a major role in FIBID of Pt from MeCpPtMe_3_ [41]. In this study, Pt(IV) reduction from Ne^+^ and Ar^+^ bombardment resulted in the loss of four CH_3_ groups, whereas He^+^ and H_2_^+^ bombardment resulted in the loss of one CH_3_ group. Deposition with the heavier ions occurred via energy/momentum transfer, while deposition with the lighter ions occurred via a combination of less efficient energy transfer and secondary electron capture. Prolonged ion exposures with heavy ions resulted in the sputtering of PtC_5_ films; however, light ion (He^+^ and H_2_^+^) irradiation resulted in very inefficient physical sputtering of Pt as compared to C atoms and therefore produced nearly pure Pt films for sufficiently high ion doses.

The present study aims to elucidate the role of precursor and ion identities in FIBID by evaluating the performance of Pt(CO)_2_Cl_2_ and Pt(CO)_2_Br_2_ as Pt precursors and comparing the differences in H_2_^+^-, He^+^-, and Ar^+^-induced Pt deposition. To determine precursor and ion identity effects on the deposition mechanism, we employ a surface analysis approach under UHV conditions to determine the ion-induced transformations of precursor thin films. In this approach, the precursor is adsorbed onto a cooled substrate to form 1–2 nm thin films. The effects of ion beam exposure on the thin films are characterized by X-ray photoelectron spectroscopy to identify changes in the films’ composition and chemical environment, and mass spectrometry to identify the volatile species produced by ion beam irradiation. Insights from these studies have been used to rationalize the composition of thin films formed by ion-induced deposition of Pt(CO)_2_Cl_2_ in the presence of Ar^+^ irradiation under steady-state deposition conditions where a substrate at room temperature is exposed to a constant flux of Pt(CO)_2_Cl_2_ molecules and Ar^+^ ions.

## Results

**Figure 1:**
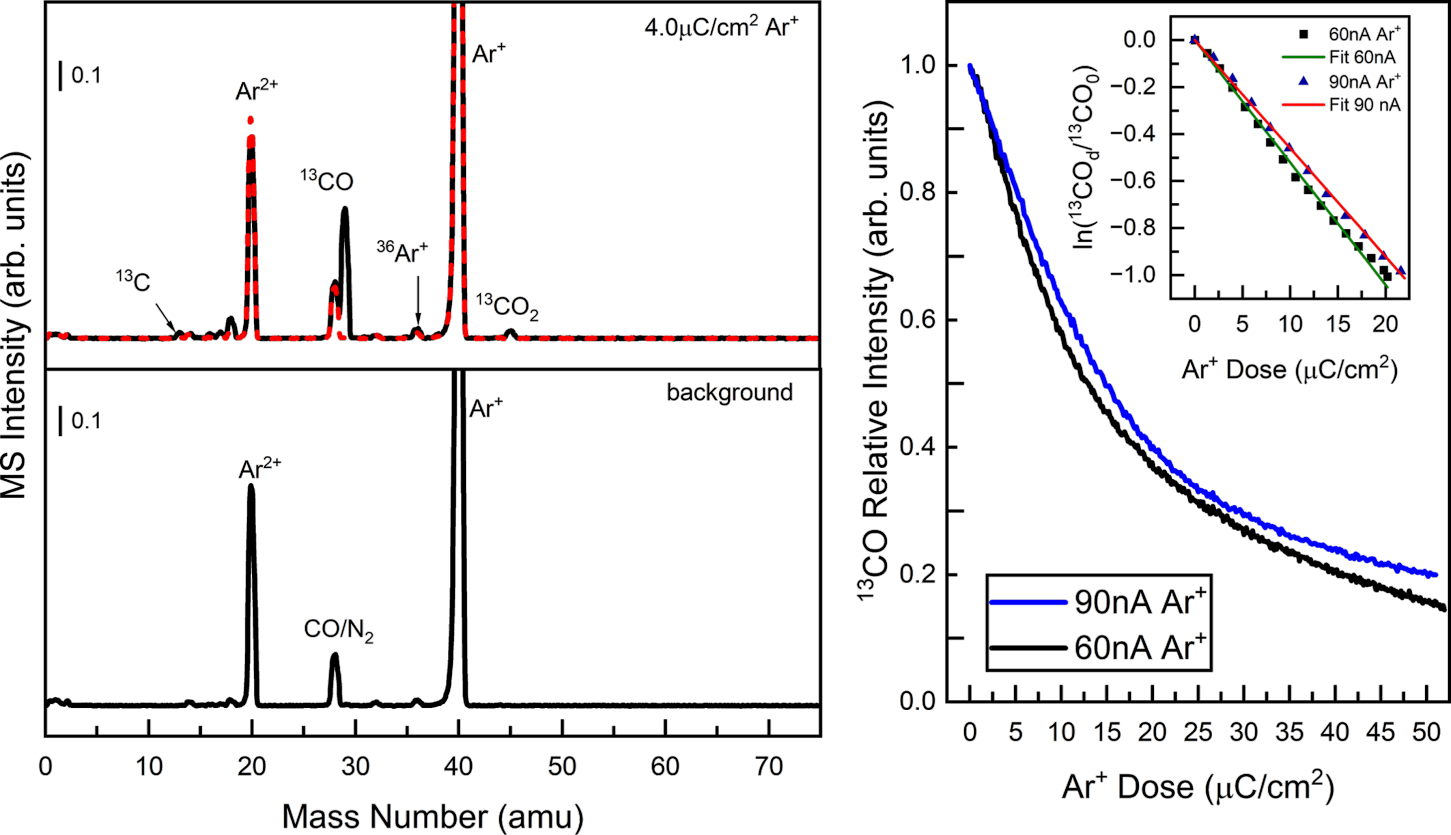
(Left) Mass spectra of the desorption products observed during ion beam irradiation of Pt(^13^CO)_2_Cl_2_. The film was irradiated with a 3.0 kV Ar^+^ beam at a flux of 100 nA. The bottom panel indicates the gas composition of the UHV chamber with 1.5 × 10^−7^ Torr Ar prior to irradiation (the peaks at *m*/*z* 40 and *m*/*z* 20 correspond to, respectively, Ar^+^ and Ar^2+^ ions formed by electron impact ionization of neutral Ar in the QMS). The top panel displays the gas phase species observed during the film’s exposure to 4.0 µC/cm^2^ Ar^+^, corresponding to an acquisition time of 30 s. (Right) Kinetic profile for ^13^CO production observed at *m*/*z* 29 for two different ion fluxes. The inset shows the linearization of the kinetic decay profiles with the corresponding fits for a first-order decay process.

The mass spectra in Figure 1 identify the volatile species produced from Ar^+^ ion irradiation of Pt(^13^CO)_2_Cl_2_. The UHV chamber background gases (H_2_, H_2_O, and ^12^CO/N_2_) and Ar (Ar^2+^, *m*/*z* 20; Ar^+^, *m*/*z* 40) are present in the bottom (black) spectrum of the left panel. The top (black) spectrum of the left panel was acquired during the initial (4.0 µC/cm^2^) exposure of the Pt(^13^CO)_2_Cl_2_ film to Ar^+^ at 3.0 kV corresponding to a 30 s acquisition time of the QMS. A new peak appears at *m*/*z* 29 along with the presence of trace peaks at *m*/*z* 13 and *m*/*z* 45, corresponding to ^13^CO, ^13^C, and ^13^CO_2_, respectively. Contributions from the background gases are superimposed (dashed red spectrum). The peaks that appear due to ion irradiation can be easily identified because of the presence of ^13^C from Pt(^13^CO)_2_Cl_2_. The right panel of Figure 1 compares the rate of ^13^CO evolution (measured at *m*/*z* 29) for different ion fluxes (black: 60 nA; blue: 90 nA). When ^13^CO evolution is plotted in terms of the ion dose for both fluxes, both profiles follow a first-order decay process with similar rate constants as seen in the inset.

**Figure 2:**
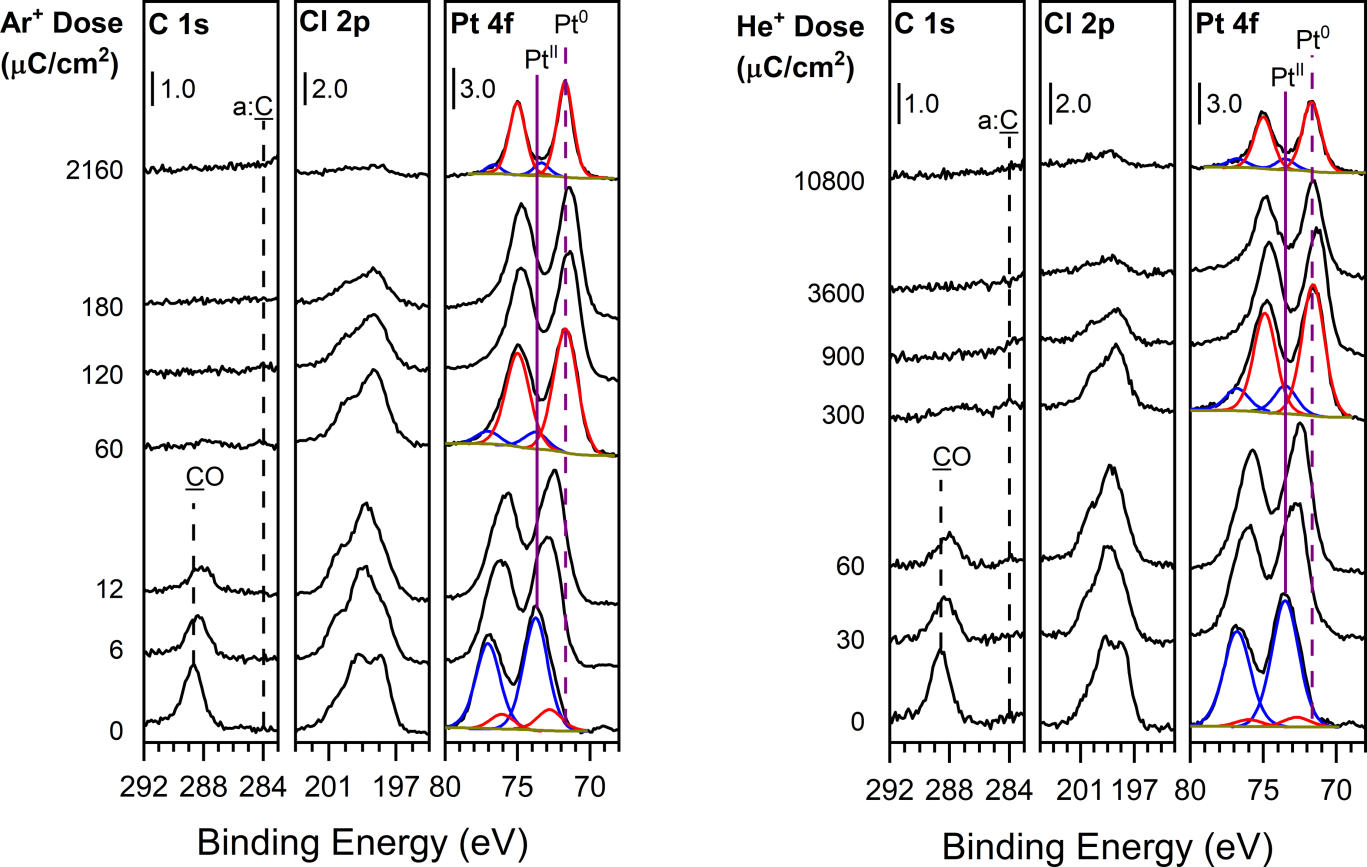
Evolution of the C 1s, Cl 2p, and Pt 4f XP regions for a thin film (≈2 nm) of Pt(CO)_2_Cl_2_ on a cooled (230 ± 5 K) tantalum oxide substrate exposed to (left) 3.0 keV Ar^+^ ions at a flux of 0.2 µA and (right) 3.0 keV He^+^ ions at a flux of 1.0 µA. In the Pt 4f region, simulated peaks are shown for the (Pt(4f_7/2_/4f_5/2_)) Pt(II) doublet associated with the parent precursor (blue) and the (Pt(4f_7/2_/4f_5/2_)) Pt(0) doublet associated with the Pt species produced as a result of ion irradiation (red). The solid and dashed purple lines indicate the binding energies for the Pt 4f_7/2_ peaks associated with the Pt(II) and Pt(0) parent and product species, respectively.

The photoelectron spectra in Figure 2 display the C 1s, Cl 2p, and Pt 4f transitions of ≈2 nm thin films of Pt(CO)_2_Cl_2_ adsorbed at 230 K as a function of increasing ion dose (bottom to top). On the left-hand side the effect of Ar^+^ irradiation is shown, while on the right-hand side the corresponding changes due to He^+^ irradiation are shown. Upon ion beam exposure, the C 1s peak at 289 eV associated with CO species [57,65] decreases in intensity with a shift towards 288 eV. For Ar^+^ exposures all of the CO is removed after an ion dose of 60 µC/cm^2^, while for He^+^ the CO peak is completely removed after an ion dose of ≈300 µC/cm^2^. Figure 2 shows that there is no evidence of amorphous carbon (a:C) being produced during ion irradiation of Pt(CO)_2_Cl_2_ by either Ar^+^ or He^+^. The Cl 2p doublet is initially centered at 199 eV, corresponding to Cl in metal chlorides, and remains in the same position while broadening with increasing ion exposure. In the initial precursor film, the position of the Pt 4f_5/2_ and Pt 4f_7/2_ peaks are at 77 and 74 eV, respectively, and are attributed to Pt(II) from the Pt(CO)_2_Cl_2_ precursor [57]. Ion exposure results in a new set of Pt 4f_5/2_ and Pt 4f_7/2_ peaks appearing at 73 and 71 eV, respectively. This shift indicates Pt reduction from Pt(II) to a Pt species that resembles Pt(0) [66–67]. As the ion dose increases, the intensity of these two new Pt peaks increases, while the intensity of the Pt(II) peaks associated with the parent compound decreases. At an Ar^+^ dose of 2160 µC/cm^2^ and a He^+^ dose of 10800 µC/cm^2^, the Pt species detected is composed entirely of Pt(0). For more prolonged ion doses in excess of these values, the Pt 4f intensity associated with the Pt(0) species formed during ion irradiation slowly decreases (data not shown).

**Figure 3:**
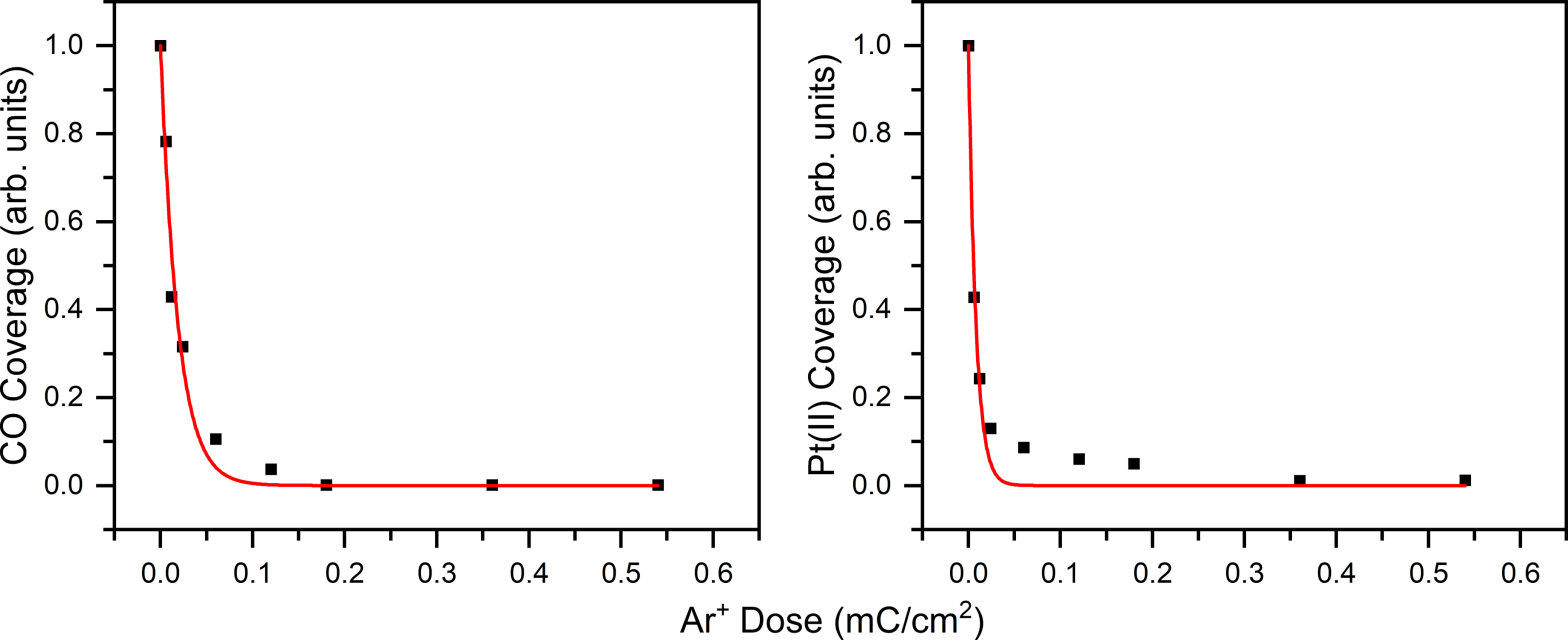
A comparison of the CO loss (left) and Pt(II) reduction (right) from Pt(CO)_2_Cl_2_ as functions of the Ar^+^ dose. The fits to the data based on a first-order kinetic process are shown in red. No further changes to C 1s and Pt(II) are observed after a 0.7 mC/cm^2^ Ar^+^ dose because the reaction has reached completion.

Figure 3 shows a comparison of the CO loss and Pt(II) reduction from Pt(CO)_2_Cl_2_ as functions of the Ar^+^ dose. Both data sets are fit to a first-order kinetic decay profile producing very similar reaction cross sections of 8.5 × 10^−15^ and 1.9 × 10^−14^ cm^2^, respectively.

**Figure 4:**
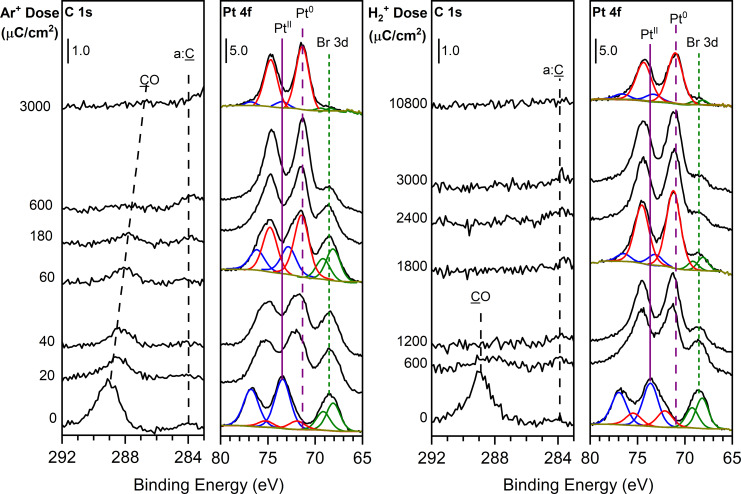
Evolution of the C 1s and Pt 4f XP regions for a thin film of Pt(CO)_2_Br_2_ on a cooled (230 ± 5 K) tantalum oxide substrate exposed to (left) 3.0 keV Ar^+^ ions at a flux of 2.0 µA and (right) 3.0 keV H_2_^+^ ions at a flux of 2.0 µA. In the Pt 4f region, simulated peaks are shown for the Pt(II) (4f_7/2_/4f_5/2_) doublet in the parent precursor (blue), the Pt(0) doublet in the deposition product (red), and the Br 3d doublet (green). The purple solid and dashed lines indicate the binding energy of the Pt 4f_7/2_ peak in Pt(II) and Pt(0), respectively, and the green dotted line indicates the peak binding energy of the Br (3d_5/2_/3d_3/2_) peaks.

Figure 4 shows how the photoelectron spectrum of Pt(CO)_2_Br_2_ evolves in response to (Figure 4, left) Ar^+^ and (Figure 4, right) H_2_^+^ exposure. Prior to ion irradiation, the C 1s region displays a single peak at 289 eV corresponding to the carbonyl (CO) groups in Pt(CO)_2_Br_2_. Upon ion irradiation, the C 1s peak decreases in intensity and shifts to 288 eV until the peak disappears at Ar^+^ and H_2_^+^ doses of 600 and 1200 µC/cm^2^, respectively. In the Pt 4f region, the Pt 4f_5/2_, Pt 4f_7/2_, and Br 3d peaks appear at 77, 74, and 69 eV, respectively, prior to ion exposure. The Pt peaks at 77 and 74 eV correspond to Pt(II) in the parent compound, and the Br peak at 69 eV corresponds to Br in metal bromides. Prior to ion irradiation, the Pt 4f peaks were found to contain a small component associated with the reduced form of Pt, as indicated by the component fit at 72 eV. The reduced Pt peak observed prior to ion irradiation is likely a consequence of the effects of X-ray irradiation that occurred during the acquisition of a full survey X-ray photoelectron spectrum from 0 to 1100 eV. Analogous to the behavior observed in Figure 2, the Pt 4f envelope broadens and shifts to lower binding energy compared to the parent peaks as a function of increasing ion dose. The change to the Pt 4f envelope that occurs upon ion irradiation can be described by contributions from the parent Pt(II) species and a new Pt species whose binding energy is similar to that of Pt(0) at 71 eV [66–67]. As the Ar^+^ and H_2_^+^ doses increase, the contribution from the parent Pt(II) species decreases until at ion doses of 3000 and 10800 µC/cm^2^, respectively, only Pt(0) peaks are observed. For these ion doses, Figure 4 shows that the film is composed almost exclusively of Pt with only trace amounts of Br present in the film.

**Figure 5:**
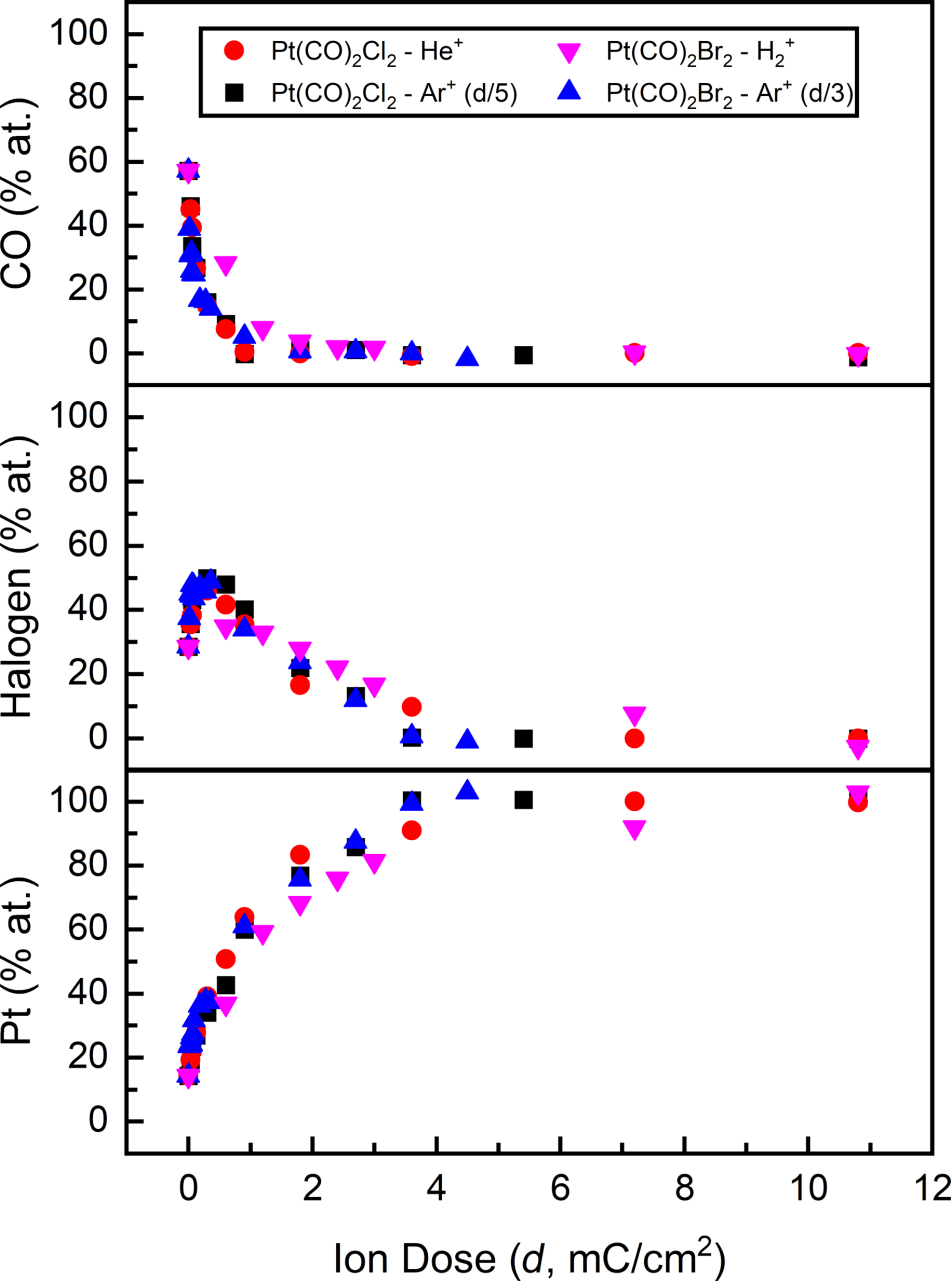
Film composition as a function of (scaled) ion dose, shown in terms of the relative concentration of CO (top), halogen (middle), and Pt (bottom). Note, that the ion doses for Pt(CO)_2_Cl_2_/Ar^+^ and Pt(CO)_2_Br_2_/Ar^+^ have been decreased by factors of 5 and 3, respectively as compared to Pt(CO)_2_Cl_2_/He^+^ and Pt(CO)_2_Br_2_/H_2_^+^.

Using the integrated area under the C 1s, Cl 2p, Br 3d, and Pt 4f regions, Figure 5 presents the atomic concentration of CO, Cl, Br, and Pt in Pt(CO)_2_Cl_2_ and Pt(CO)_2_Br_2_ films during ion irradiation. The initial concentration was normalized to the precursors’ (C+O)/X/Pt atomic stoichiometry of 4:2:1, which corresponds to 58 atom % CO, 28 atom % X (Cl or Br), and 14 atom % Pt prior to ion irradiation (*d* = 0 mC/cm^2^). It should be noted that for comparative purposes the ion dose has been scaled for different precursor/ion combinations; specifically, the ion doses for Pt(CO)_2_Cl_2_/Ar^+^ and Pt(CO)_2_Br_2_/Ar^+^ have been decreased by factors of 5 and 3, respectively, as compared to Pt(CO)_2_Cl_2_/He^+^ and Pt(CO)_2_Br_2_/H_2_^+^. Analysis of Figure 5 reveals that trends in film composition amongst all of the different precursor/ion combinations as a function of the ion dose are remarkably similar. This strongly suggests that the elementary reaction steps involved in the ion beam-induced reactions are invariant to the specific ion/precursor, although the relative rates of these individual steps are larger for Ar^+^.

It is evident from Figure 5 that at comparatively low ion doses (<1 mC/cm^2^), the CO concentration rapidly decays, consistent with the desorption of CO observed in Figure 1. This leads to a corresponding increase in the fractions of Pt and halogen in the films (Figure 5). For higher ion doses, XPS data (Figure 2) shows that both halogen and Pt atoms are removed from the film, but the rate of halogen removal is noticeably faster. Consequently, after a (scaled) ion dose of ca. 4 mC/cm^2^ only Pt is present. It should be noted that although these precursors yield nearly pure Pt films, they are less than 0.5 nm thick as compared to the initial film thicknesses of ≈1.3 nm, largely as a consequence of Pt sputtering that occurs before all of the halogen atoms are removed, which occurs at ≈1 mC/cm^2^ in Figure 5.

**Figure 6:**
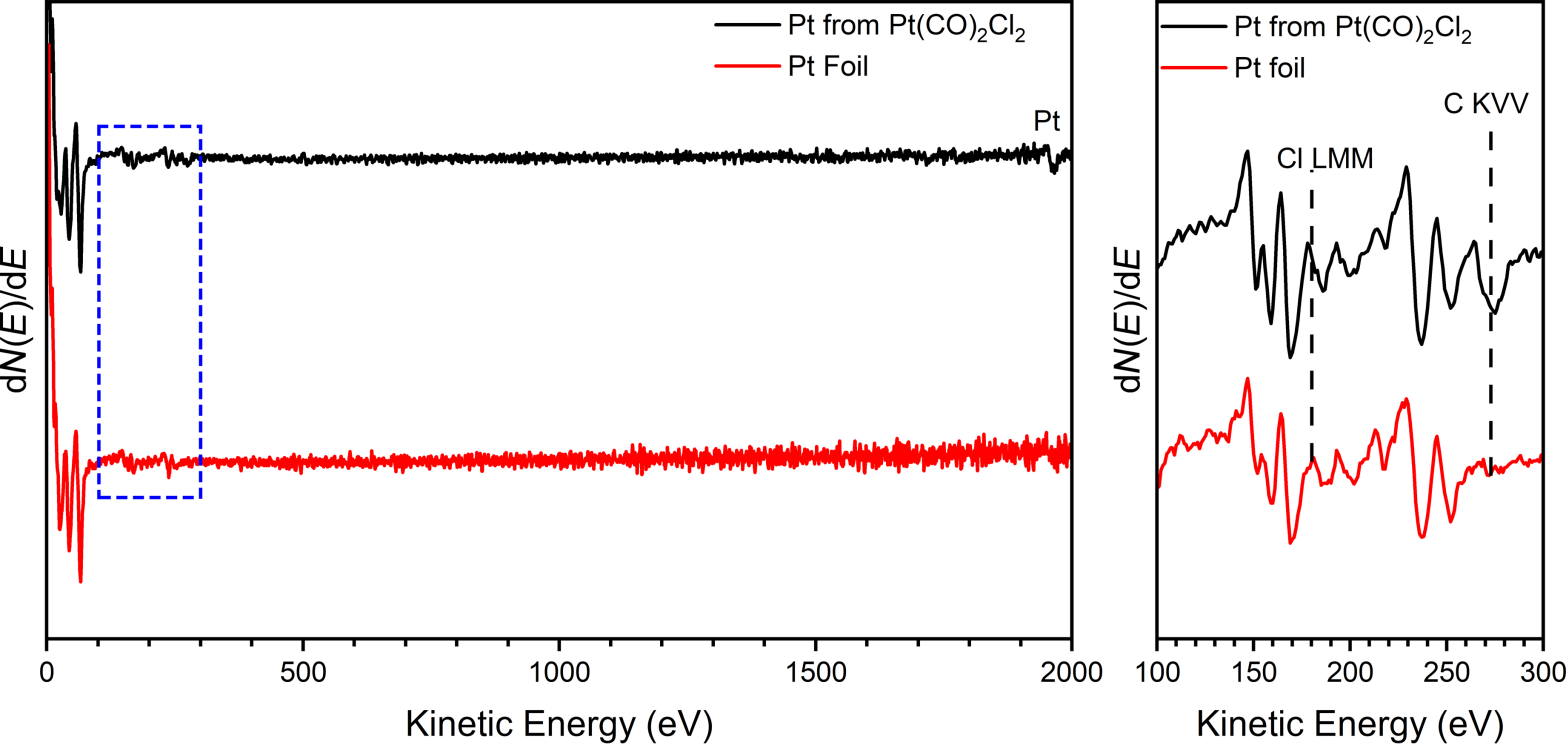
(Left) AES survey spectra collected at 5 keV for Pt foil (red trace) and a deposit from Pt(CO)_2_Cl_2_ produced under steady-state conditions (black trace); full details are provided in the Experimental section. (Right) Detailed spectrum in the low-energy region. The dashed lines denote the kinetic energy positions of Cl LMM and C KVV Auger transitions.

Figure 6 and Figure S5 (Supporting Information File 1) show the results of experiments where a Si substrate was exposed to a constant partial pressure of Pt(CO)_2_Cl_2_ and a steady Ar^+^ flux of 5 nA at an incident energy of 800 eV. These conditions represent a situation that describes the typical deposition of structures by IBID, one where a substrate is exposed to a constant partial pressure of the precursor in the presence of simultaneous ion irradiation. Initial experiments resulted in Pt deposits that were not sufficiently thick to analyze with XPS or AES. To address this issue, the ion beam was rastered over a 5 × 5 mm^2^ area, and the deposition time was increased. Deposits created in this way were found to be thick enough to be analyzed ex situ by AES (Figure 6) and XPS (Figure S5, Supporting Information File 1).

Figure 6 shows the AES data of a film created under these conditions after surface contaminants (carbon and oxygen) that unavoidably accrued as a result of sample transfer from the deposition chamber to the AES system were removed by Ar^+^ sputtering. This data is shown alongside the AES data of a sputter-cleaned Pt foil measured in the same chamber. This comparison reveals that the deposit created from Pt(CO)_2_Cl_2_ is almost identical to the pure Pt reference, with only a trace level of carbon contamination and no evidence of oxygen or chlorine. The analogous XPS data is shown in Figure S5 (Supporting Information File 1) where the as-deposited film shows no evidence of any chlorine although carbon and oxygen are observed. We ascribe the presence of carbon and oxygen to be in part due to the adsorption of background CO in the growth chamber onto the exposed Pt surface as it was warmed to room temperature. Light Ar^+^ sputtering significantly reduces the concentration of both carbon and oxygen signals and leads to a concomitant increase in the Pt signal. It should also be noted that the Pt 4f peak position of 71.0 eV observed by XPS is virtually identical to that of the new Pt(0) Pt 4f peak position observed during ion irradiation of Pt(CO)_2_Cl_2_ films deposited at low temperature and exposed to ion irradiation (Figure 2).

## Discussion

MS and XPS data support the idea that, upon ion beam exposure, the initial process to occur is Pt–CO bond dissociation, evolving both CO ligands as the first volatile product. The volatilization of CO is readily identified in Figure 1 where ion irradiation of isotopically labelled Pt(^13^CO)_2_Cl_2_ yields a mass spectrum with an intense peak at *m*/*z* 29 corresponding to ^13^CO. Furthermore, CO loss follows a first-order decay process with respect to ion dose, with a rate constant that is independent of ion flux. The photoelectron spectra in Figure 2 and Figure 4 support the MS data that for each precursor/ion pair, complete CO desorption is the initial step in the reaction because the peaks in both the C 1s and O 1s regions are lost during the initial stages of ion irradiation and at comparable rates. Concurrent with the CO desorption, the Pt 4f peaks shift (2–3 eV) toward lower binding energy, which is indicative of an increase in electron density around the Pt center associated with Pt reduction from Pt(II) (B.E. = 74 eV) to a state closer to metallic (i.e., Pt(0); B.E. = 71 eV). Moreover, the rate of Pt(II) reduction is correlated with the rate of CO loss (Figure 3). Collectively, these observations point towards an initial step that can be described by Equation 1:


[1] $$\begin{array}{l} {\text{Pt(CO)}}_{2}{\text{X}}_{2}\;\left(\text{ads}\right)+{\text{Z}}^{+}\left(\text{g}\right)\overset{\sigma_{1}}{\to }{\text{PtX}}_{2}\left(\text{ads}\right)+2\text{CO}\left(\text{g}\right).\\ \left(\text{Z}={\text{H}}_{2},\text{He},\text{Ar}\right) \end{array}$$

The preferential loss of CO is attributed to the stability of CO as a volatile species and the relatively lower Pt(II)–CO bond dissociation energy in the coordination complex compared to the stronger ionic interaction between Pt(II) and the halide ligands. The increasing Pt and halogen fractions in the films as functions of ion irradiation, as seen in Figure 5, are a consequence of the reaction described in Equation 1. During the CO loss process, the C 1s peak associated with the metal carbonyl decreases in intensity and shifts (1 eV) towards lower binding energy, an effect that can be attributed to an increase in the electron density around the metal center, as we have observed in previous studies [24,56–57,68]. The halogen peaks broaden somewhat, indicating a more heterogeneous local environment as the deposit forms from the molecular precursor, although the invariant peak position indicates that there is little change in the average chemical environment surrounding the halogen atoms.

Since the loss of CO from all of the Pt(CO)_2_X_2_ films can be described by a first-order kinetic process, we can extract a reaction cross section σ_1_ for each ion/precursor combination studied. This is illustrated in Figure S3 (Supporting Information File 1) by following the change in the relative intensity of the C 1s photoelectron peak as a function of ion dose. Results of this analysis are tabulated in Table 1.

**Table 1:** Reaction cross sections for Pt(CO)_2_X_2_ dissociation.

Precursor/Ion Pair	σ_1_ (×10^15^ cm^2^)

Pt(CO)_2_Cl_2_/He^+^	1.6 ± 0.2
Pt(CO)_2_Cl_2_/Ar^+^	8.5 ± 0.3
Pt(CO)_2_Br_2_/Ar^+^	3.8 ± 0.5
Pt(CO)_2_Br_2_/H_2_^+^	0.35 ± 0.03
Pt(CO)_2_Cl_2_/e^−^ (500 eV)^a^	0.15

^a^Spencer et al. [57]

Ar ion-induced decomposition of surface-adsorbed organometallic precursors has previously been attributed to momentum transfer from the ion to the adsorbed precursor as a result of inelastic nuclear collisions [21–23,25,41]. Given the smaller mass of H_2_^+^ and He^+^ compared to Ar^+^, we would anticipate significantly less efficient momentum/energy transfer from these lighter ions to the much heavier Pt(CO)_2_X_2_ precursors because of the extremely poor mass match in these cases. Furthermore, heavier ions have shallower interaction volumes than lighter ions, increasing the likelihood of their collisions with the adsorbed precursor molecules [37,41]. These two factors are primarily responsible for the larger reaction cross section observed for Ar^+^ irradiation compared to He^+^ or H_2_^+^ (Table 1). Table 1 also shows that the σ_1_ value for CO desorption from Pt(CO)_2_Br_2_/H_2_^+^ is proximate to the σ_1_ value that we have previously measured for the electron-induced CO desorption from Pt(CO)_2_Cl_2_ [57]. The relative proximity of these two σ_1_ values suggests that the reactivity of H_2_^+^ ions may contain a significant contribution from reactions involving the low-energy electrons produced by ion–substrate interactions. In this respect, the increased importance of this reaction pathway is a consequence of the extremely inefficient momentum transfer for H_2_^+^/Pt(CO)_2_X_2_ collisions, coupled with the greater penetration depth of the lighter ions within the tantalum/tantalum oxide substrate, which would favor the production of secondary electrons. These findings also mirror results from a recent study involving the effect of ion identity on reactions involving another Pt precursor, MeCpPtMe_3_ [41].

Following CO desorption, the next step involves the physical sputtering of halogen and Pt atoms from the PtX_2_ species created by CO loss. Experimentally this is evidenced by the decrease in halogen content in the film while the Pt fraction in the deposit increases (Figure 5):


[2] $$\begin{array}{l} {\text{PtX}}_{2}\left(\text{ads}\right)+{\text{Z}}^{+}\to \text{Pt}\left(\text{ads}\right)+\text{X}\left(\text{g}\right).\\ \left(\text{Z}={\text{H}}_{2},\text{He},\text{Ar}\right) \end{array}$$

It should be noted that although the change in the film’s composition indicates that the loss of halogen atoms is the preferential sputtering step, the decrease in the Pt 4f signal over this same ion dose regime demonstrates that Pt atoms are being lost at the same time through sputtering, albeit at a slower rate.

Although Figure 5 shows that the same sequence of reactions (CO desorption followed by physical sputtering of halogen atoms at a faster rate than Pt) occurs regardless of the precursor/ion combination, there is a significant difference in the absolute sputter rates. For example, in Figure 2 and Figure 4 an Ar^+^ dose of 2160 µC/cm^2^ results in complete Cl removal from PtCl_2_, whereas a 3000 µC/cm^2^ Ar^+^ dose was required to completely remove Br from PtBr_2_. Since ion beam-induced physical sputtering is driven by a momentum/energy transfer mechanism with a dependence on the mass ratio between the ion and target [69–72], this difference can attributed to the better mass match between Ar^+^ and PtCl_2_/Cl as opposed to the heavier PtBr_2_/Br as well as the higher velocity imparted to Cl species in any energy transfer process. Similarly, the ion dose required for Cl and Br removal from PtX_2_ species using the much lighter He^+^ (4 amu) and H_2_^+^ (2 amu) ions is substantially higher (7200 µC/cm^2^ for both ions) than the corresponding Ar^+^ doses (2000–3000 µC/cm^2^).

Once all of the halogen atoms have been removed, a nearly pure Pt film remains. The nearly pure Pt film is itself, as stated above, still susceptible to sputtering. This is evidenced experimentally in XPS by a decrease in film thicknesses for ion doses in excess of the values needed to create pure Pt films:


[3] $$\begin{array}{l} \text{Pt}\left(\text{ads}\right)+{\text{Z}}^{+}\to \text{Pt}\left(\text{g}\right).\\ \left(\text{Z}={\text{H}}_{2},\text{He},\text{Ar}\right) \end{array}$$

Thus, the overall sequence of elementary reaction steps, initiated by CO desorption as a consequence of momentum/energy transfer from the incident ion to the precursor, followed by preferential physical sputtering of halogen atoms before the residual metal atoms are ultimately removed by physical sputtering, is analogous to the ion-induced reactions with adsorbed Ru(CO)_4_I_2_ [23].

In the present study, the relative efficiency of Pt atom sputtering is expected to be greatest for the heavier Ar^+^ ions [37,41]. Indeed, other related studies have shown that Pt atom sputtering is extremely inefficient for H_2_^+^ and He^+^ ions [41]. In the context of FIBID, this means that, although He^+^ and H_2_^+^ will require the largest ion doses to produce pure Pt films, once the films have been formed, they will be more resistant to the unwanted effects of physical sputtering, ultimately affording the possibility to form thicker Pt films albeit at slower rates and requiring higher ion/precursor ratios.

These elementary reaction steps that accompany FIBID can also be compared to the reactions of Pt(CO)_2_Cl_2_ with electrons studied under similar UHV conditions, representing the other type of charged particle used to effect deposition of metal-containing structures from organometallic precursors. Electron-induced Pt deposition from Pt(CO)_2_Cl_2_ was reported to follow an initial step similar to that in ion-induced reactivity with the desorption of CO and the formation of PtCl_2_, although in the case of electron irradiation this initial step was ascribed to dissociative electron attachment as opposed to momentum transfer [57]:




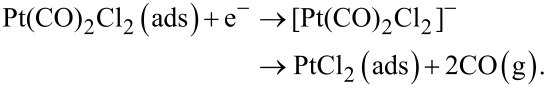




At much higher electron doses, Cl was removed by electron stimulated desorption to convert PtCl_2_ to Pt:




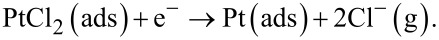




However, to achieve complete Cl desorption, an electron dose of approximately 1000 mC/cm^2^ was required [57]. In comparison, He^+^- and Ar^+^-induced deposition requires, respectively, ion doses of 7.2 and 2.1 mC/cm^2^ to effect complete Cl removal from the PtCl_2_ film. This highlights the vastly more efficient removal of halogen atoms from PtX_2_ intermediates by means of ion-induced sputtering as compared to electron-stimulated desorption.

In comparison to a previous study involving MeCpPtMe_3_ [41], the role of the incident ion is less pronounced for Pt(CO)_2_X_2_ precursors, with all of the ion/precursor combinations following the same elementary sequence of reaction steps. This can be rationalized by the facile loss of CO groups during the deposition step involving Pt(CO)_2_X_2_ precursors, in contrast to the greater energetic demands associated with the removal of Pt–CH_3_ ligands from MeCpPtMe_3_. As a result, the elementary reaction steps are invariant to the ion identity for Pt(CO)Cl_2_ and Pt(CO)_2_Br_2_ with the role of the incident ion identity being restricted to a kinetic effect.

The previous sections have described changes to the composition of PtX_2_(CO)_2_ films exposed to various inert gas ions and the rationale for these transformations. Although these studies demonstrate that Pt(CO)_2_X_2_ films could be used to create pure Pt films during IBID, these assertions are based on data obtained under UHV conditions with a thin adsorbate film molecularly adsorbed onto an inert substrate and exposed to inert gas ions. To demonstrate that these elementary reaction steps can rationalize the behavior of films deposited from Pt(CO)_2_X_2_ precursors under steady-state deposition conditions, we conducted the experiments described in Figure 6 as well as Figure S4 and Figure S5 (Supporting Information File 1). The AES data in Figure 6 and the XPS data in Figure S5 (Supporting Information File 1) reveal that no detectable Cl or C signals are present in the Ar^+^-induced Pt deposits. In contrast, electron-induced deposition of Pt from Pt(CO)_2_Cl_2_ was found to contain impurities depending on the background environment. Notably, electron-induced deposits formed under UHV conditions contained 58% Cl and 37% Pt with no detectable C or O signatures in EDX [55,57]. The AES and XPS data shown in Figure 6 as well as Figure S4 and Figure S5 (Supporting Information File 1) support the conclusions of the UHV studies, specifically that Pt(CO)_2_X_2_ can be used as a precursor for creating pure Pt films during FIBID. However, using Pt(CO)_2_X_2_ as precursors for depositing nanostructures by means of FIBID will require gas injection systems that can be heated sufficiently to maintain a reasonable precursor partial pressure during deposition. Thus, the AES and XPS data are reported for films that are certainly less than 50 nm thick and which required many hours of deposition time to create.

## Conclusion

Low-energy ion irradiation of adsorbed Pt(CO)_2_Cl_2_ and Pt(CO)_2_Br_2_ initiates complete CO desorption as a result of ion/molecule energy transfer, leading to a reduction of the parent Pt(II) species and the creation of adsorbed PtCl_2_ or PtBr_2_ species. Additional ion irradiation results in physical sputtering and the removal of halogen atoms and, simultaneously, a slower removal of Pt. The preferential sputtering of the residual halogen atoms produces a pure Pt film. Although the rates of ion-induced CO desorption and physical sputtering of Pt and halogen atoms depended on the ion identity (Ar^+^, H_2_^+^, or He^+^), the sequence of elementary reaction steps involved in the ion-induced reactions of Pt(CO)_2_Cl_2_ or Pt(CO)_2_Br_2_ was common to all of the ion/precursor combinations. This sequence of elementary reaction steps identified under UHV conditions for Pt(CO)_2_Cl_2_ and Pt(CO)_2_Br_2_ films adsorbed at low temperatures also rationalizes our observation that Ar^+^ ion beam-induced deposition of Pt(CO)_2_Cl_2_ can create pure Pt films under steady-state deposition conditions typical of IBID.

## Experimental

### Precursor synthesis

#### General synthesis procedure

All reactions were carried out under an inert atmosphere of dinitrogen using either Schlenk or glovebox techniques. Glassware was flame-dried or oven-dried before use. Solvents (i.e., dichloromethane (DCM, CH_2_Cl_2_), 1,2-dichloroethane (DCE, C_2_H_4_Cl_2_), toluene, and *n*-heptane) were purified using an MBraun MB-SP solvent purification system and stored over 3 Å molecular sieves before use. All reagents were purchased from Millipore Sigma and used without further purification. Carbon monoxide (CP grade), and ^13^C carbon monoxide were either purchased from Airgas or Millipore Sigma. Deuterated solvents for NMR were purchased from Cambridge Isotopes Lab and were stored over 3 Å molecular sieves in a glovebox prior to use. ^13^C NMR spectra were obtained on a Bruker 400 MHz spectrometer. All chemical shifts are reported in δ (ppm) and were referenced to the solvent. IR spectra were obtained on a PerkinElmer Spectrum ONE FTIR spectrometer using a solution cell equipped with NaCl windows and a path length of 1.0 mm.

#### *cis*-Pt(CO)_2_Br_2_

The compound was synthesized using a modified literature procedure [73]. In a glove box, a glass pressure vessel was charged with PtBr_2_ (0.31 g, 0.88 mmol), a magnetic stir bar and dry DCE (30 mL). The glass pressure vessel was placed in a 300 mL Parr reactor for 2 h at room temperature under CO (150 psi). The temperature was then increased to 110 °C by a sand bath, and the reaction mixture was stirred for another 3 h. After the reactor was cooled to room temperature, the reaction mixture was stirred overnight. The unreacted CO gas was then released, and the reactor was backfilled with N_2_. The Parr reactor was brought into the glove box and opened inside. The DCE solvent and the yellow-brown suspension were transferred into a Schlenk flask inside the glove box. The Schlenk flask was moved to a Schlenk line, and the DCE solvent was removed under vacuum to leave a yellow-brown powder inside the flask. The flask was brought into the glove box again and a cold finger was added. After purifying the yellow-brown crude compound by sublimation at 30–35 °C at 125 ± 1 mTorr, a light-yellow solid (0.28 g, yield 80%) was obtained. ^13^C NMR (CDCl_3_, 500 MHz) δ 152.34 (^1^*J*_C–Pt_ = 1546 Hz); IR (CH_2_Cl_2_, Figure S1, Supporting Information File 1): ν_co_ 2129, 2170 cm^−1^.

#### *cis*-Pt(CO)_2_Cl_2_

The compound was prepared according to a literature procedure [57]. Platinum(II) iodide (0.10 g, 0.22 mmol) was suspended in 15 mL of toluene in a 50 mL Schlenk flask. After bubbling CO into the suspension for 2 h, the color of the solution changed from black to brown, and then sulfuryl chloride (0.15 g, 1.1 mmol) was added to the solution. The reaction mixture was stirred for 6 h until the color of the solution changed to dark purple. Then 15 mL of anhydrous *n*-heptane was added to the reaction mixture, and it was kept overnight in the freezer, which finally resulted in the formation of pale white crystals. The solvent was removed by cannula transfer, and the solid was washed with *n*-heptane until all the purple color was gone. Then the product was dried under vacuum for 6 h, which resulted in the formation of needle-like crystals of *cis-*Pt(CO)_2_Cl_2_ (0.04 g, yield 56%). ^13^C NMR (C_6_D_6_, 400 MHz) δ 151.15; IR (toluene, Figure S1, Supporting Information File 1): ν_co_ 2128, 2170 cm^−1^_._

#### *cis*-Pt(^13^CO)_2_Cl_2_

Platinum(II) iodide (0.30 g, 0.66 mmol) was suspended in 25 mL of toluene in a 100 mL Schlenk flask and was stirred under closed conditions maintaining 1 atm pressure of ^13^CO for 2 h. The color of the solution changed from black to brown, and then sulfuryl chloride (0.45 g, 3.30 mmol) was added to the solution. The reaction mixture was stirred for 6 h until the color of the solution changed to dark purple. Then 25 mL of anhydrous *n*-heptane was added to the reaction mixture, and it was kept overnight in the freezer, which finally resulted in the formation of pale white crystals. The solvent was removed by cannula transfer, and the solid was washed with *n*-heptane until all the purple color was gone. The product was dried under vacuum for 6 h, which resulted in the formation of needle-like crystals of *cis-*Pt(^13^CO)_2_Cl_2_ (0.10 g, yield 47%). The product sublimes at 35–40 °C at 125 mTorr. ^13^C NMR (C_6_D_6_, 400 MHz) δ 151.71 (^1^*J*_C–Pt_ = 1562 Hz); IR (toluene, Figure S1, Supporting Information File 1) ν_co_: 2077, 2118 cm^−1^.

### UHV studies

Experiments were performed in a stainless-steel ultrahigh vacuum system as described elsewhere [21]. Briefly, a cooled tantalum/tantalum oxide substrate (230 ± 5 K) was exposed to Pt(CO)_2_Cl_2_ or Pt(CO)_2_Br_2_ vapor (ca. 5.0 × 10^−8^ Torr) for approximately 30 min to produce 1–2 nm thin films of each precursor. Pt(CO)_2_Cl_2_(s) was heated to 80–85 °C, and Pt(CO)_2_Br_2_(s) was heated to 85–90 °C to ensure sufficient volatility for thin film growth, as performed previously [55,57]. The Pt(CO)_2_Cl_2_ films were exposed to Ar^+^ (3.0 kV; 0.2 µA) and He^+^ (3.0 kV; 1.0 µA) ions, and the Pt(CO)_2_Br_2_ films were exposed to Ar^+^ (3.0 kV; 2.0 µA) and H_2_^+^ (3.0 kV; 2.0 µA) ions.

XPS was used to monitor changes in the films’ composition and the Pt oxidation state. Photoelectron spectra were collected using a PHI 5400 XPS equipped with a 300 W Mg Kα X-ray source at an analyzer pass energy of 22.36 eV. The spectra were analyzed using CasaXPS [21,41,74], a commercially available analysis software. Synthetic peak fitting was used in the Pt 4f region to monitor changes in the Pt oxidation state. Peak fitting for the Pt 4f and Br 3d peaks was performed with 60/40 Gaussian/Lorentzian line shapes, GL(40), in CasaXPS [21,57–58].

Mass spectrometry, using a Pfeiffer QMS 200 mass analyzer, was used to identify the evolved species resulting from ion irradiation. Isotopically labeled Pt(^13^CO)_2_Cl_2_ was used in mass spectrometry experiments to distinguish between ^13^CO (*m*/*z* 29) liberated during ion irradiation and ^12^CO/^14^N_2_ (*m*/*z* 28) in the UHV chamber background.

### Steady-state deposition

Ion beam deposits produced under steady-state conditions were produced in a separate UHV chamber equipped with a Stanford Research Instruments RGA200 quadrupole mass spectrometer and a PHI 04-303 ion gun. The base pressure of this chamber was 2.9 × 10^−9^ Torr. Si substrates (20 × 20 mm, Ted Pella, Inc.) were cleaned by sonication in acetone, then isopropanol, and finally blown dry with 99.999% N_2_ (Airgas) and used as the substrate for deposition. Approximately 500 mg of Pt(CO)_2_Cl_2_ (Strem Chemicals) was loaded into a glass vial that was attached to a UHV precision leak valve. A 1/8″ stainless steel directional dosing line was attached to the vacuum side of the leak valve to maximize the precursor pressure at the target surface. The directional dosing line, the leak valve body, and the precursor reservoir were resistively heated to 70 °C to volatilize the precursor and prevent condensation on the steel components. The leak valve was fully opened during deposition for maximum precursor flux, corresponding to an increase in chamber pressure of 5.3 × 10^−8^ Torr. Ion beam-induced deposition of Pt(CO)_2_Cl_2_ was performed using the ion gun operating at a constant pressure of 99.999% Ar (Airgas) and an incident energy of 800 eV and target current of 5 nA. The 0.8 mm ion beam was rastered over a 5 × 5 mm area. Chamber gas composition, Ar purity, and precursor pressure at the beginning of the deposition were monitored by mass spectrometry. Ion irradiation was conducted for 18.5 h to provide sufficiently thick deposits for subsequent analysis.

The composition of the resulting deposits was analyzed ex situ using a PHI 5600 XPS with a Mg Kα X-ray source at the Johns Hopkins University and a PHI 660 Scanning Auger Multiprobe at an incident electron energy of 5 keV at the University of Florida. AES data was also compared to sputter cleaned Pt foil (99.9% Beantown Chemical).

## Supporting Information

FTIR spectra of Pt(^12^CO)_2_Cl_2_, Pt(^13^CO)_2_Cl_2_, and Pt(CO)_2_Br_2_, evolution of film contrast as a function of Ar^+^ dose, the decay of the C 1s X-ray photoelectron intensity as a function of Ar^+^, He^+^, and H_2_^+^ dose, XPS survey of Pt deposits formed under steady-state conditions, and detailed XPS spectra of deposits formed under steady-state conditions.

File 1 Additional experimental data.

## Data Availability

All data that supports the findings of this study is available in the published article and/or the supporting information to this article.
