# Supplementary material for: Ion-induced surface reactions and deposition from Pt(CO)2Cl2 and Pt(CO)2Br2
Source: Beilstein J Nanotechnol. 2024 Nov 19;15:1427–39. doi: 10.3762/bjnano.15.115 (PMC11590011; doi:10.3762/bjnano.15.115)
Supplement: File 1 — Additional experimental data. [file Beilstein_J_Nanotechnol-15-1427-s001.pdf]

BEILSTEIN JOURNAL OF NANOTECHNOLOGY

Supporting Information

for

Ion-induced surface reactions and deposition from $Pt(CO)_2Cl_2$ and $Pt(CO)_2Br_2$

Mohammed K. Abdel-Rahman, Patrick M. Eckhert, Atul Chaudhary,
Johnathon M. Johnson, Jo-Chi Yu, Lisa McElwee-White and D. Howard Fairbrother

Beilstein J. Nanotechnol. **2024,** *15,* 1427–1439. doi:10.3762/bjnano.15.115

Additional experimental data

License and Terms: This is a supporting information file under the terms of the Creative Commons Attribution License (https://creativecommons.org/licenses/by/4.0). Please note that the reuse, redistribution and reproduction in particular requires that the author(s) and source are credited and that individual graphics may be subject to special legal provisions.
The license is subject to the *Beilstein Journal of Nanotechnology* terms and conditions: (https://www.beilstein-journals.org/bjnano/terms)

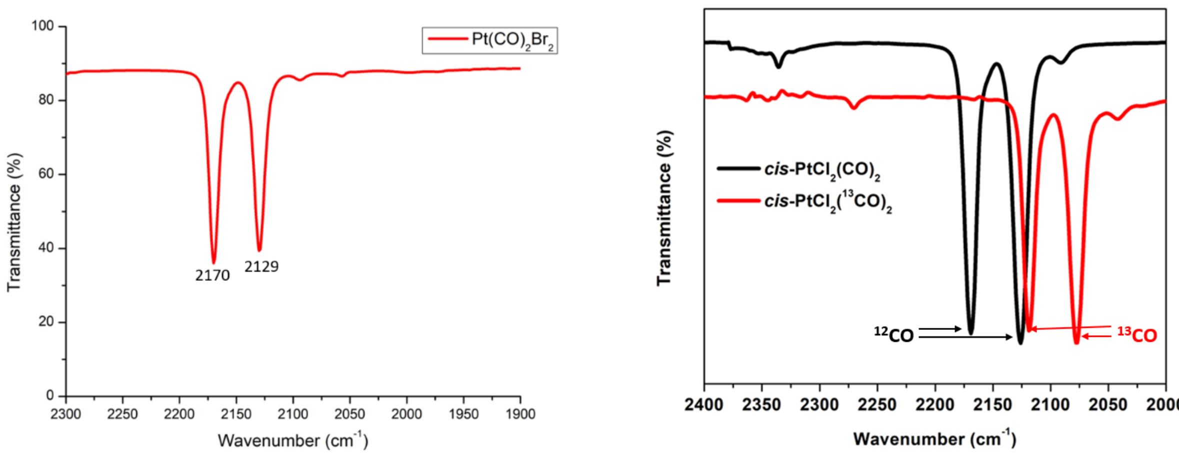

Figure S1: (Left) FTIR spectrum of *cis*-Pt(CO)$_2$Br$_2$ obtained in dichloromethane solution. (Right) FTIR spectra of *cis*-Pt(^{13}CO)$_2$Cl$_2$ (red) and *cis*-Pt(CO)$_2$Cl$_2$ (black) obtained in toluene solution.

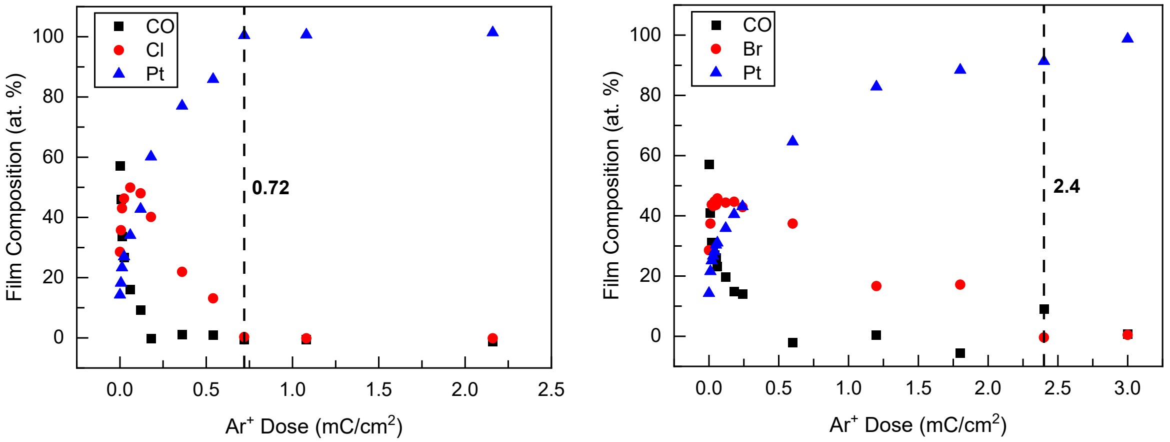

Figure S2: Film composition as a function of Ar^{+} ion exposure to Pt(CO)$_2$Cl$_2$ (left) and Pt(CO)$_2$Br$_2$ (right) as determined by XPS. The dotted line represents the minimum dose required to achieve a pure Pt film.

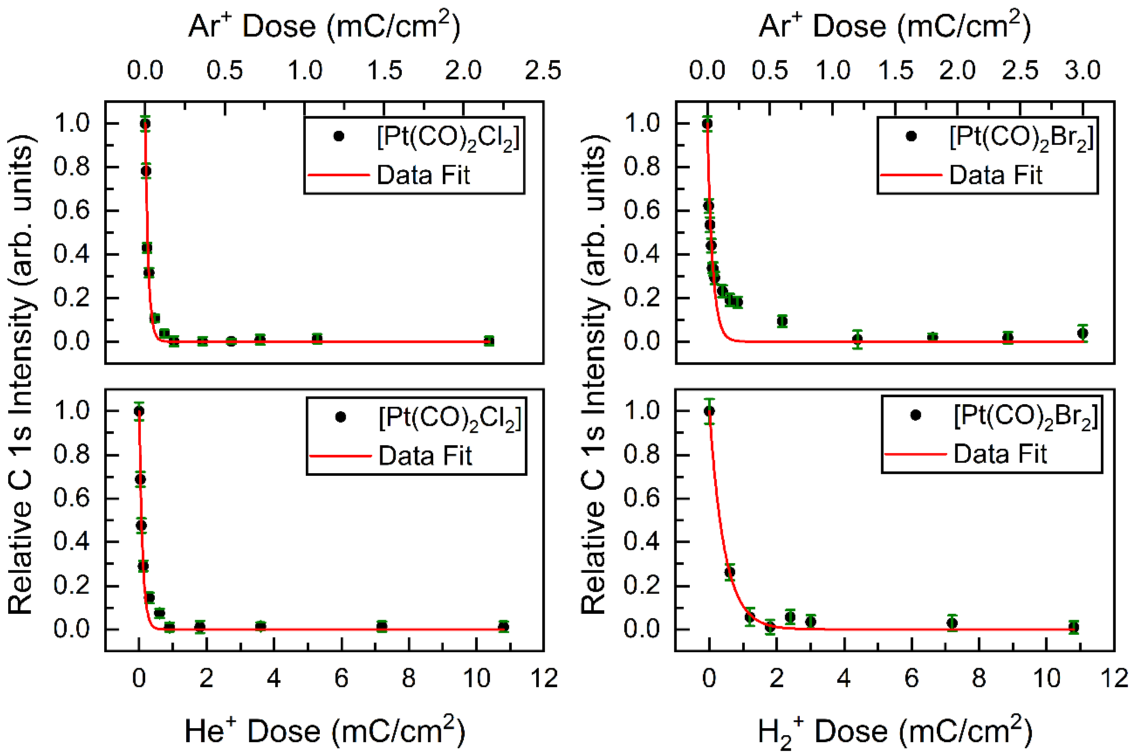

Figure S3: Change in carbon XPS intensity as a function of ion dose for each ion/precursor pair. The red line corresponds to a fit to a first-order kinetic decay profile.

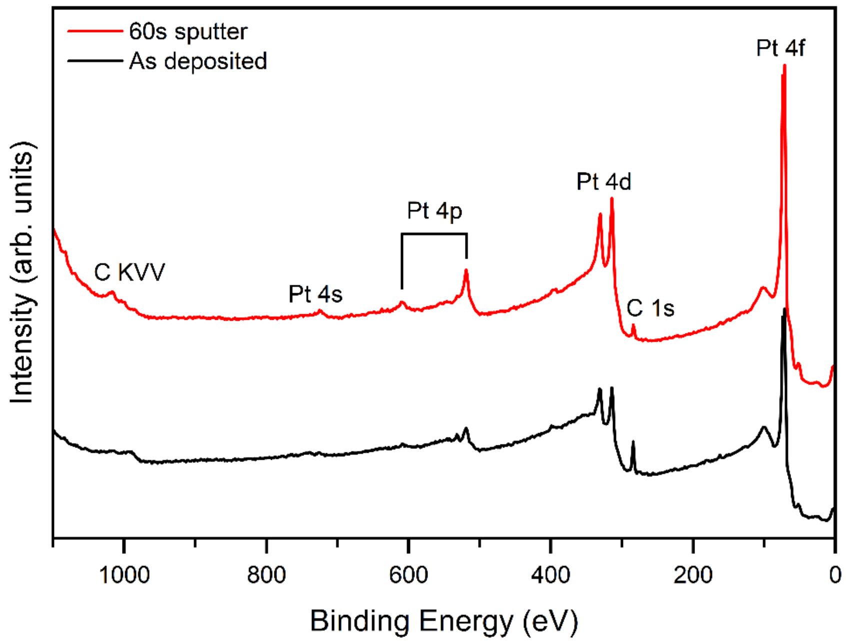

Figure S4: XPS survey spectra of a steady-state $Pt(CO)_2Cl_2$ deposit before (black) and after (red) a 60 s 1 kV Ar^+ sputter to remove adventitious carbon.

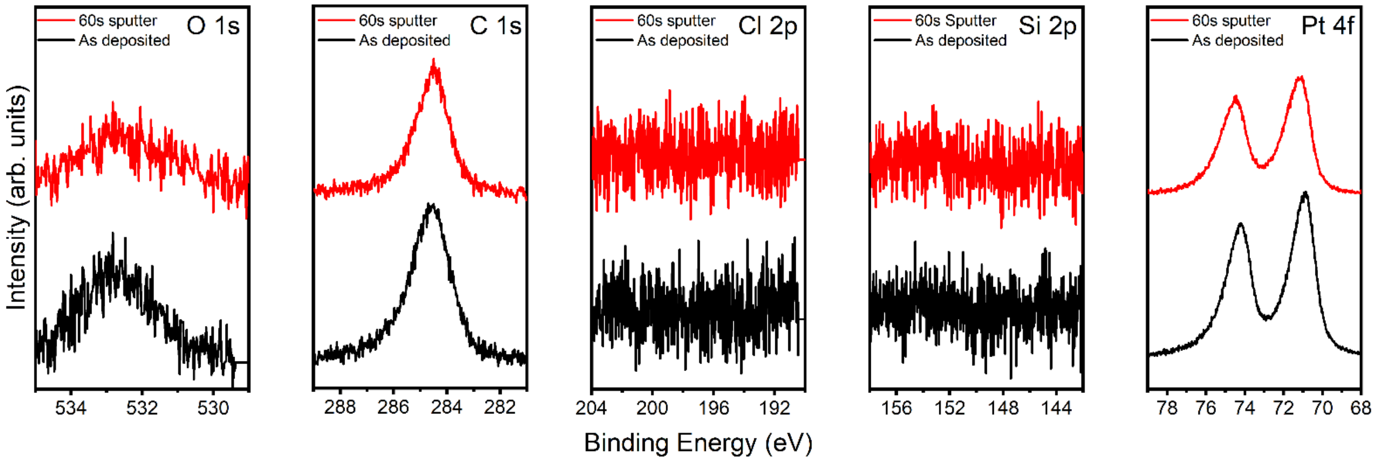

Figure S5: XPS spectra of the O (1s), C (1s), Cl (2p), Si (2p), and Pt (4f) regions of a steady-state $Pt(CO)_2Cl_2$ deposit before (black) and after (red) a 60 s 1 keV Ar^+ sputter to remove adventitious carbon.
